# Fiscal vertical imbalance and income inequality: A threshold effect analysis based on government expenditure

**DOI:** 10.1371/journal.pone.0317537

**Published:** 2025-01-24

**Authors:** Lan Mao

**Affiliations:** School of Public Finance and Taxation, Southwestern University of Finance and Economics, Chengdu, Sichuan, China; Palembang City Government, INDONESIA

## Abstract

This study aims to investigate the complex relationship between vertical fiscal imbalance, public expenditure structure, and income distribution disparities, with the goal of providing policy insights for achieving shared prosperity. Employing Generalized Method of Moments (GMM) and threshold analysis, the research reveals key findings: (1) an exacerbation of vertical fiscal imbalance significantly widens the urban-rural income gap; (2) public expenditure structure exhibits threshold effects, resulting in non-linear impacts on income disparity; (3) a unique contribution of our study is the identification of varying threshold effects of urban public expenditure on income disparities within rural areas, urban areas, and the gap between them, underscoring the need for targeted fiscal interventions. These findings highlight the critical role of public expenditure in addressing income distribution issues and offer valuable guidance for upcoming fiscal and tax reforms.

## 1. Introduction

### 1.1. Background

The Fifth Plenary Session of the 19th Central Committee of the Communist Party of China (CPC) has underscored the imperative to “actively foster common prosperity.” This objective resonates with the Party’s governance ethos and the popular will, representing the fundamental purpose of economic and social progress: ensuring prosperity for all citizens. Since the inception of the reform and opening-up policy, China has experienced a remarkable surge in its economic and comprehensive national strength. However, challenges persist, including significant disparities in urban-rural development and income distribution, as well as issues of unbalanced and inadequate development [1]. The substantial income inequality and unfair distribution mechanisms have emerged as critical impediments to China’s transition through the “middle-income trap” [2].

In response to these challenges, the CPC Central Committee, under the leadership of General Secretary Xi Jinping, has identified the need to establish fundamental institutional frameworks for aligning initial, secondary, and tertiary distribution, guiding the next phase of fiscal and tax reforms. This emphasis on harnessing the institutional strengths of fiscal policy in aligning income distribution has emerged as a pivotal strategy for advancing communal wealth and achieving societal harmony and stability. Nonetheless, the prevailing fiscal structure in China, which is rooted in the 1994 tax-sharing reform, confronts two significant challenges moving forward.

From a vertical perspective, there is an uneven distribution of revenue rights and expenditure obligations between the central and local governments, resulting in a disparity between their respective authorities and financial resources [3]. This imbalance has objectively contributed to the growing problem of local fiscal constraints. From a horizontal viewpoint, local governments exhibit structural distortions in their fiscal revenue and expenditure patterns [4]. The urgent task at hand is to determine the optimal approach for restructuring public expenditures to boost the input efficiency of fiscal spending, thereby improving income distribution in China [5, 6].

### 1.2. Research questions and significance

Hence, examining the moderating effect of fiscal instruments on income distribution necessitates an analysis of the influence of the fiscal characteristics of vertical disequilibrium between central and local governments on residential income disparity, along with an assessment of the role of the structure of local fiscal expenditures [7].

Given the aforementioned background, this study aims to address the following research questions: How does vertical fiscal imbalance (VFI) affect the income disparity among residents? What is the role of the local fiscal expenditure structure in mediating income distribution? Does the threshold effect of government spending vary by region? How do different categories of expenditures influence income distribution among urban dwellers, the income disparities between rural residents, and the income gap between urban and rural residents? The study seeks to provide a comprehensive understanding of the complex relationship between fiscal behavior and income distribution within China.

The significance of this research lies in its contribution to the theoretical understanding of the relationship between fiscal policies and income distribution in the context of China’s unique economic and social landscape. It provides empirical evidence that can inform policy decisions aimed at reducing income disparities and promoting more equitable fiscal practices. It offers insights into the optimization of public expenditure structures, which is crucial for achieving the goal of common prosperity.

### 1.3. Research objectives

This research aims to: firstly, analyze how vertical fiscal imbalance between central and local governments affects residential income disparity; secondly, evaluate the role of local fiscal expenditures in shaping income inequality among urban and rural residents; furthermore, explore the threshold effects of various government expenditure categories on income distribution; and finally, provide empirical evidence and policy recommendations for optimizing public expenditure structures to advance the goal of common prosperity in China.

### 1.4. Research innovations

The innovative aspects of this study can be summarized as follows: Firstly, it conducts an empirical analysis demonstrating that longitudinal fiscal imbalances not only exacerbate income disparities within rural areas, urban areas, and the gap between them but also exhibit homogeneity within these groups. This finding complements the existing literature on income inequality research within urban-rural dual economic structures, particularly through the use of the Gini coefficient. Secondly, the study examines the structure of government expenditures to analyze the income gap between urban and rural residents, delving into the threshold effects of different government expenditure categories. This approach offers a fresh research perspective on the interplay between fiscal policy and income distribution. Finally, the conclusions provide a quantitative assessment of the implementation efficiency of public expenditures, offering empirical support for improving local fiscal relations and reshaping equitable and efficient mechanisms since the tax-sharing reform.

### 1.5. Research approach and strategy

The research strategy encompasses the following steps: Firstly, indices are constructed to accurately gauge income disparity among residents and the degree of vertical fiscal imbalance faced by local governments. Secondly, the Generalized Method of Moments (GMM) model is employed to analyze the effects of vertical fiscal imbalance in local finances on the income disparity between urban and rural residents following China’s tax-sharing reform. Furthermore, recognizing the complexity of the transmission mechanism between vertical fiscal imbalance and income inequality, a threshold model is utilized for rigorous testing and estimation, considering the direct influence of the structure of public expenditures. Finally, the study strategically selects Gini coefficient data from 28 provinces over the period 1995 to 2019, a timeframe that captures the profound effects of the 1994 tax reform, excludes the distortions of the COVID-19 pandemic, and encompasses multiple complete economic cycles, thereby facilitating a comprehensive analysis of long-term trends and cyclical effects of fiscal policies.

### 1.6. Organization of the paper

The remainder of the paper is structured as follows: The second part is dedicated to theoretical analysis and the establishment of research hypotheses. The third part provides an explanation of the data and the methodology for measuring indicators. The fourth part delves into empirical analysis. The fifth section succinctly summarizes the main findings and police recommendations, limitations and uncertainties, and future work plans.

## 2. Literature review

### 2.1. Vertical fiscal imbalance and fiscal decentralization theory

Vertical fiscal imbalance (VFI), characterized by a mismatch between local governments’ revenue and expenditure responsibilities, underscores the broader issue of financial resource allocation between central and local authorities [8, 9]. This imbalance has significant implications for wealth distribution, sparking debates on the optimal extent of fiscal autonomy for local governments [10, 11]. Despite extensive discourse, a consensus remains elusive [12, 13]. Theoretical perspectives on fiscal decentralization, such as those proposed by Tiebout [9] and Oates [8], suggest inefficiencies in local income redistribution post-decentralization [14, 15]. Conversely, McKinnon [16] and others [17, 18] argue for the significant role of local governments in income redistribution, with Sacchi [19] emphasizing the need for substantial fiscal autonomy [20].

### 2.2. Government expenditure behavior and income inequality in China

With the increasing demand for public expenditure in China, there is a growing body of research aimed at identifying the most effective methods to ascertain the optimal scale and structure [21]. Domestic research primarily integrates with the behavior of local governments, offering a nuanced understanding of the fiscal landscape [22, 23]. Liu Dan et al. [3] argue that the increase in central-local shared expenditures and expenditures that should not be borne by local governments are significant reasons for the intensification of vertical imbalance in China’s fiscal system in recent years. This misalignment between expenditure and income responsibilities is associated with the “public pool problem” [24, 25]. The promotion of local government officials, linked to GDP growth and tax revenue performance [26, 27], creates incentives for prioritizing economically generative expenditures over public welfare, thereby widening the urban-rural income gap [28, 29].

### 2.3. Threshold effects and nonlinear relationships in fiscal policy

While numerous studies have examined China’s local fiscal issues, few directly link these to income disparities among residents [30, 31]. Chen Anping [32] highlights the impact of fiscal policies on the urban-rural income gap. A study [33] introduce a novel approach by examining the variable effects of vertical fiscal asymmetry on income inequality through a transfer payment threshold model, which differs from traditional regression analysis as noted by [34, 35].

This study’s focus on the threshold effect of public expenditure structure in the context of vertical fiscal imbalance represents a departure from standard regression analysis, as noted by the research [36]. Another study [37] also investigated the threshold effect of transfer payments on the relationship between fiscal decentralization and income inequality, using a different threshold variable than the present study.

### 2.4. International comparisons and policy insights

Although the primary focus is on China, international research offers valuable insights into issues such as optimal tax structures, the efficiency of social welfare expenditures, and the decision-making processes of different levels of government. For example, Ara et al. [38] investigated the relationship between local public goods provision in Pakistan and local self-generated income, highlighting the importance of local fiscal autonomy. Eyraud and Lusinyan [39] examined the fiscal implications of VFI reductions in various countries, showcasing diverse approaches to addressing fiscal imbalances. Researchers [40] provide insights from Russia’s fiscal decentralization reform. Another study [41] discussed the influence of financial autonomy on income distribution in Bulgaria. Additional perspectives are offered from Thailand and Poland [42, 43].

These international comparisons are crucial for understanding the global context of fiscal policies and their impact on income distribution, offering a broader perspective for analyzing China’s fiscal system. Nonetheless, it is essential to consider the unique Chinese context [28, 44].

### 2.5. Synthesis and gaps in the literature

The literature review offers a comprehensive understanding of vertical fiscal imbalance, public expenditure structures, and their implications for income inequality. However, international studies, often rooted in Western electoral systems, may not fully capture the unique fiscal challenges faced by China [44, 45].

Domestic research, while insightful, highlights several areas that require further refinement and enrichment. Firstly, a more nuanced analysis is needed to understand how vertical fiscal imbalance specifically impacts residential income disparity in China, considering regional differences and demographic factors. Secondly, the role of local fiscal expenditure structure in mediating income distribution demands further empirical investigation, particularly in light of China’s rapid economic development and urbanization trends. Thirdly, the exploration of threshold effects and nonlinear relationships within different categories of government expenditure remains limited and necessitates deeper analysis. These gaps highlight the need for further research within the Chinese context.

## 3. Theoretical analysis and research hypotheses

### 3.1. Vertical fiscal imbalance and urban-rural income gap

Post-1994 tax-sharing reform, China’s VFI has shifted from local exclusivity to central-local sharing, significantly impacting local tax revenues and the tax base. Local governments, constrained by budgetary debt issuance, have adopted various revenue-generating strategies, including increased debt, infrastructure investment, and land sales. These strategies, driven by inter-regional competition and financial constraints, have prioritized high-yield short-term investments, exacerbating fiscal deficits. In response, local governments have implemented measures such as tax rate adjustments and enhanced public goods provision, which have inadvertently affected the income disparity between urban and rural residents.

Research identifies several channels through which VFI affects the urban-rural income gap: Firstly, urban-biased fiscal investment within the vertical structure of fiscal resource allocation leads local governments to prioritize the allocation of limited fiscal resources to urban areas [46]. Such urban-biased fiscal policies result in insufficient investments in rural areas in terms of infrastructure and public services.

Second, imperfections in the transfer payment system fail to effectively compensate for the fiscal deficiencies in rural areas in practice [29, 46]. The inadequacy of transfer payments leads to a lack of public service provision in rural areas, which not only intensifies welfare disparities between urban and rural areas but also further expands the urban-rural income gap.

Third, the unequal distribution of educational resources and opportunities means that urban areas typically possess superior educational resources, medical facilities, and transportation convenience, in contrast to rural areas, which often face a relative lack of these amenities. Educational inequality, as a crucial driver of the widening urban-rural income gap, is increasingly evident [47]. Research [48] indicates that the disparity in educational levels accounts for 34.69% of the urban-rural income gap. This unequal distribution of educational resources and opportunities constrains the employment prospects and income growth potential of rural residents.

Additionally, studies have investigated the effects of vertical fiscal imbalance on various economic and social aspects, including public expenditure structure [49], land finance [50], the efficiency of social spending [29], and total factor productivity [51]. While these studies do not directly address the urban-rural income gap, they shed light on how vertical fiscal imbalance can indirectly influence the gap through its impact on these factors. Therefore, the paper puts forward Hypothesis 1.

**H1:** An increase in the fiscal imbalance between the central and local governments will contribute to a widening of the income disparity among residents.

### 3.2. Public expenditure structure and income gaps

The inadequate and uneven provision of public goods is a key challenge in the country’s current development, characterized by insufficient spending and excessive structural investments. As noted [52], the primary principles guiding the vertical relations between Chinese governments in public goods supply are economic efficiency and social risk. Public spending influences the natural movement of factors and the redistribution of resources, altering residents’ disposable income and thus bridging income gaps and safeguarding social welfare. Conversely, it also examines the social risks arising from local governments’ short-term profit-seeking in public expenditure decisions.

Given China’s unique centralized system, local governments exhibit a “risk communal pot” behavior, with fiscal risks exhibiting a highly divergent institutional nature. Extensive financing in construction shifts “contingent liability” risks to subsequent governments [24]. Moreover, the “promotion tournament” among horizontal governments may fuel local governments’ short-term profit-seeking behavior [50].

Evidently, under the VFI system, the income distribution effect of local fiscal spending has a dual nature of positive improvement and concurrent risks. Hence, the paper proposes Hypothesis 2:

**H2:** The structures of public expenditure have threshold effect, leading to a nonlinear relationship between vertical fiscal imbalance and the income gap.

The economic development between urban and rural areas in China exhibits a pronounced imbalance, mirrored in the allocation of public expenditures. This disparity has been a persistent issue since the initial divergence of urban and rural economies. Urban areas, benefiting from favorable locations and resource distribution, have seen swift economic growth. This growth, coupled with higher living standards, has led local governments to focus more on the quality of economic development. In contrast, rural areas, hampered by various constraints, lag in economic development, making it their primary concern.

Additionally, public spending by local governments is influenced by the central government’s evaluation of their economic performance. Local officials often show little interest in public spending that doesn’t significantly boost economic indicators, resulting in poor public service quality in rural areas.

The dynamic changes in public spending structure also reflect the level of fiscal imbalance. Different public spending initiatives have varying impacts on income distribution, and the same initiative can affect urban and rural incomes differently. For instance, social security spending can offer rural residents with lower incomes and worse living conditions more financial security, narrowing the urban-rural income gap. Moreover, spending on science, education, culture, and health is crucial in addressing poverty’s intergenerational transmission and has a positive effect on human capital, employment, and income growth. However, unequal educational resource distribution and the crowding-out effect between urban and rural areas can widen the income gap.

In the subsequent empirical section of our study, we delineate the income disparities not only between urban and rural residents but also within these respective groups, extending beyond the scope of Hypothesis 2 which solely addresses the urban-rural income gap. Consequently, we propose Hypothesis 3:

**H3:** Distinct categories of public expenditure exhibit a threshold effect, resulting in a non-linear correlation between vertical fiscal imbalances and the income disparities among urban residents, as well as between such imbalances and the income gaps among rural residents.

## 4. Variable definition and data description

### 4.1. Explained variables

Conventional Gini coefficient measurements aren’t a good fit for China’s unique dual economic structure. Globally, the Gini coefficient is the go-to metric for assessing income inequality, but it fails to account for the distinct income disparities within urban and rural populations, or the vast income chasm between these two groups. To bridge this gap, China has enhanced its research methods with proxy indicators, primarily using a comprehensive Gini coefficient that incorporates the Theil index, the income difference between urban and rural residents, and a breakdown of urban-rural disparities. This paper follows the existing research methodologies [37, 53], calculating the Gini coefficients for urban and rural residents’ incomes using formula (1).


$$G=1-\frac{1}{ZP}\sum_{i=1}^{n}\left(Z_{i-1}+Z_{i}\right)P_{i}$$ (1)

In formula (1), *P* denotes the total urban (rural) resident population at year-end, while *Z* signifies the total income of urban (rural) residents. The subscript i indicates the number of groups categorized by income level, with *P*_*i*_ representing the population of the i-th group and *Z*_*i*_ representing the cumulative income of that group. We have manually compiled the income data for each province into five groups using the quintile approach. By ascertaining the population and income of each group, the specific value of the Gini coefficient can be determined. It is evident that the calculation process involves grouping residents based on income levels, yet it does not enforce stringent rules regarding the grouping methodology. This aspect is crucial for calculating the Gini coefficient in scenarios involving unequally partitioned income data.

After calculating the Gini coefficient for the income of urban and rural residents, we proceed to apply group-weighted method [54], as detailed in formula (2), to ascertain the overall Gini coefficient for the income of residents throughout China’s provinces.


$$P_{u}^{2}\frac{R_{u}}{R}G_{u}+P_{r}^{2}\frac{R_{r}}{R}G_{r}+P_{u}P_{r}\frac{R_{u}-R_{r}}{R}$$ (2)

In formula (2), *R* stands for the per capita income of each province, which is the Gini coefficient for the entire province’s residents. The subscript *u* refers to urban areas, and *r* to rural areas. *G*_*u*_ (*G*_*r*_) shows the Gini coefficient for urban (rural) residents’ income. *P*_*u*_ (*P*_*r*_) is the population proportion of urban (rural) residents. *R*_*u*_ (*R*_*r*_) is the per capita income of urban (rural) residents. For the calculation of the median of the opening group in the income grouping interval, the method is: the median of the missing lower (upper) limit group = interval upper (lower) limit ± (adjacent group interval / 2) [55].

Since Shanghai didn’t release income grouping data for the whole city from 2015 to 2017, without distinguishing between urban and rural areas, the Gini coefficient remained the same as in 2014. Also, it’s worth mentioning that some provinces have missing data on residents’ income. We calculated the Gini coefficients for urban, rural, and overall residents’ income across 28 provinces in China, excluding Jilin Province, Shandong Province, Taiwan Province, Tibet Autonomous Region, and the special administrative regions of Hong Kong and Macau from 1995 to 2019.

### 4.2. Core explanatory variables

#### 4.2.1 Vertical fiscal imbalance

Vertical fiscal imbalance (VFI) is identified by the mismatch between the financial authority and administrative responsibilities of local governments, resulting in an imbalance between their revenues and expenditures. Post the tax-sharing reform, which entailed the centralization of financial power at the central government and the decentralization of administrative authority to local governments, a natural gap in the vertical fiscal asymmetry at the local tier was formed. The conventional approach to measure VFI involves constructing the local fiscal self-sufficiency gap. Although some literature has proposed more complex quantitative methodologies for VFI, the majority of empirical research continues to employ the traditional measurement technique [56]. The specific construction method of the vertical fiscal imbalance indicator is detailed in Table 1.

**Table 1:** Description of the VFI construction.

Name of index	Computational formula	Variable definition
Vertical fiscal imbalance (VFI)	$\begin{array}{c} \mathrm{VFI}=1-\frac{\text{ID}}{\text{ED}}\times (1\text{-FSG}) \end{array}$	PFR: Local public budget revenue per capita PFE: Per capita local public budget expenditure CFR: Per capita central public budget revenue CFE: Per capita central public budget expenditure FR: Local public budget revenue FE: Local public budget expenditure
Decalization of fiscal revenue (ID)	$\begin{array}{c} ID=\frac{PFR}{PFR+CFE} \end{array}$	
Decalization of fiscal expenditure(ED)	$\mathrm{ED}=\frac{PFE\;}{PFE+CFE}$	
Fiscal self-sufficiency rate (FSG)	$\mathrm{FSG}=\frac{FE\text{-}FR}{FE}$	

#### 4.2.2 Structure of public expenditure

Different fiscal items have varying impacts on the urban-rural income disparity, making it crucial to differentiate these items to understand their effects on income distribution [57]. Since 2007, empirical studies in this area have either employed outdated data to tackle current issues or new data to re-examine historical ones. The former method is less convincing and accurate because of the data’s age, while the latter is limited in its descriptive power due to a constrained observation period.

In the process of selecting fiscal expenditure classification variables, this study drew on the relevant research [58–60]. In particular, it referenced the method for classifying fiscal expenditures proposed by certain scholars [58], and the empirical method established by other scholars [49] laid the foundation for the research in this article.

The data on public expenditure in this paper span from 1995 to 2019. Initially, the inflation factor was eliminated, and then the stable public expenditure items were categorized into five groups. The structure of public expenditure was reflected through the proportion of these expenditures in the total GDP. After such treatment, although there are statistical fluctuations in our data, the trend of the data remains continuous and comparable.

The detailed breakdown is as follows: Firstly, “SECHS” expenditure, which includes spending on science, technology, education, culture, sports, media, and health, has incorporated cultural, sports, and media activities since 1997. Secondly, “AFW” expenditure, covering agriculture, forestry, and related sectors, was integrated into the category of agriculture, forestry, and water conservancy after 2007. Thirdly, public safety expenditure, comprising defense, armed police, and security services, was merged into the category of defense and public safety after 2007. Fourthly, social security expenditure, which comprises pensions, welfare, retirement funds, and subsidies, was reclassified under the category of social security and employment after 2007. Finally, public administration expenditure, previously categorized as general public service, was classified as such before 2007.

### 4.3. Control variables

In the empirical model of this study, several control variables have been introduced to account for their potential impact on the income inequality between urban and rural residents. These control variables are defined as follows:

1. Economic development level (lngdp): To eliminate the influence of price factors and enhance the stability of the data, the per capita GDP of each province is index-adjusted against the base year of 1995 and then log-transformed. Regions with higher levels of economic development are expected to provide more abundant and higher-quality public goods and services, such as social security, which contribute to elevated living standards and overall income levels for the population.
2. Openness to trade (open): Adjusted for exchange rate volatility, the total import and export volume of each province is converted into Chinese Renminbi (RMB) using the annual average exchange rate and then divided by the province’s GDP for that year. The level of trade openness can significantly influence the economic conditions and income structure within a locality.
3. Government competition (govrise): This variable is represented by the ratio of actual foreign investment in each province to the total foreign investment across the nation. As local governments incur costs, including tax incentives and infrastructure investments, to attract foreign capital, the volume of foreign investment reflects the intensity of competition among local governments for resources
4. Financial development level (finance): Measured as the proportion of the financial industry’s value added to GDP in each province, financial development can enhance income levels among the poor through its impact on economic growth.
5. Physical Capital (phycap): This is represented by the ratio of fixed asset investment to GDP in each province, which indicates the region’s reliance on investment-driven economic development.
6. Human capital (humcap): This is quantified by the proportion of college students in each province relative to the total population, reflecting the impact of education on human capital and income.
7. Urbanization rate (urban): This variable is measured as the ratio of the urban resident population to the total population within each province. Urbanization levels can impact tax revenues and the provision of public goods, thereby influencing income distribution across the region.
8. Population density (density): This is computed by dividing the total population at the end of the year by the administrative area of each province. Increased population density may result in diminished marginal returns from the provision of public goods.

The raw data for all variables utilized in this study are sourced from the China Statistical Yearbook(1996–2020), Local Statistical Yearbook(1996–2020), Local Survey Yearbook(1996–2020), and the China Macro Network. absence of original data for certain provinces, the analysis is based on a sample that includes data from 28 provinces, including Beijing, Shanghai and Anhui. The data characteristics, such as mean values and standard deviations for specific variables, are presented in Table 2.

**Table 2:** Descriptive statistics.

Variable name	Variable declaration	Mean	Standard Error	Least Value	Crest Value
**Gini**	Overall Gini coefficient	0.377	0.057	0.228	0.499
**Gini** ^**u**^	Urban Gini coefficient	0.280	0.041	0.135	0.389
**Gini** ^**c**^	Rural Gini coefficient	0.307	0.050	0.110	0.469
**VFI**	Longitudinal fiscal imbalance	0.654	0.198	-0.006	0.979
**q**_**1**_	SECHS	0.048	0.025	0.010	0.142
**q**_**2**_	Agriculture	0.019	0.017	0.002	0.109
**q**_**3**_	Public safety	0.011	0.005	0.003	0.054
**q**_**4**_	Social security	0.024	0.017	0.000	0.140
**q**_**5**_	Administration	0.019	0.010	0.004	0.073
**lngdp**	Economic development level	8.584	0.498	6.074	9.940
**open**	Open to the outside world	0.300	0.378	0.013	2.051
**finance**	Financial development level	0.053	0.031	0.007	0.190
**govrise**	The government competition	0.509	0.648	0.016	5.705
**phycap**	Material capital	0.568	0.261	0.205	1.516
**hucap**	Human capital	1.507	0.564	0.377	2.824
**urban**	Urbanization rate	0.474	0.171	0.163	0.896
**density**	Density of population	7.355	0.972	1.609	8.749

## 5. Empirical analysis

### 5.1 Vertical fiscal imbalance and household income inequality

#### 5.1.1 Basic model

According to Hypothesis 1, the complex pressures resulting from vertical fiscal imbalance drive local governments to shift their focus from a "helping hand" that aims to decrease income inequality to a "grabbing hand" pursuing power and wealth [61]. This imbalance is anticipated to Expanded the income disparity among residents further. To confirm Hypothesis 1, the study employs a basic model, represented by Eq (3).


$$Gini_{it}=\partial_{0}+\partial_{1}VFI_{it}+\emptyset X_{it}+\varepsilon_{it}$$ (3)

Initially, we established a baseline model using Ordinary Least Squares (OLS) to examine the disparities among provinces, with a particular focus on the relationship between vertical fiscal imbalance and income inequality among residents. The OLS results indicated a significant positive correlation at the 1% significance level, even in the absence of controls for province-specific fixed effects. To capture the nuances of panel data and to provide a more rigorous analysis, we subsequently employed fixed effects models with province dummy variables (LSDV) and a two-way fixed effects model (FE). The Hausman test results, with a p-value below 0.001, strongly rejected the null hypothesis of the random effects model, suggesting a correlation between individual effects and the explanatory variables. This finding supported the preference for fixed effects models over random effects models, as presented in Table 3, which includes regression results from OLS, LSDV, and FE models. Control variables, such as the level of economic development, were introduced incrementally to mitigate multicollinearity concerns.

**Table 3:** Results of the base regression.

Variable	(1)OLS	(2)LSDV	(3)FE
**VIF**	0.125*** (0.030)	0.066*** (0.013)	0.066*** (0.012)
**Control Variables**	yes	yes	yes
**Individual fixed effects**		yes	yes
**Time-point fixed effects**			yes
**Observed Values**	700	700	700
**Robust(R²)**	0.668	0.817	0.583

Note: *, ** and *** are significant at 10%, 5% and 1%, respectively, with data as standard error in parentheses.

Despite the ability of fixed effects models to control for province-specific influences, they do not address potential endogeneity issues. To this end, we adopted the Instrumental Variable Two-Stage Least Squares (IV-2SLS) method. We selected instrumental variables based on established practices, including lagged terms of vertical fiscal imbalance, degree of openness, government competition, level of economic development, and the Gini coefficient. The two-stage results of the IV-2SLS model are displayed in Table 4.

**Table 4:** Two-stage least squares (2SLS) instrumental variable method regression results.

Phase one regression	VFI	lngdp		open		govrise
**L.VFI**	0.848*** [0.029]	-0.113** [0.044]		-0.055* [0.030]		-0.200* [0.106]
**L.lngdp**	-0.018* [0.010]	0.844*** [0.015]		0.035*** [0.011]		-0.029 [0.037]
**L.open**	-0.055*** [0.014]	0.231** [0.021]		0.904*** [0.014]		-0.009*** [0.050]
**L.govrise**	0.002 [0.005]	0.051 [0.044]		0.009* [0.005]		0.843*** [0.018]
**F-value**	555.01	1406.22		2085.31		735.3
**Two-stage regression**	**(4) Overall Gini coefficient**		**(5) Urban Gini coefficient**		**(6) Rural Gini coefficient**	
**VFI**	0.176*** [0.018]		0.106*** [0.018]		0.131*** [0.025]	
**lnndp**	-0.043*** [0.006]		0.017*** [0.006]		0.025*** [0.009]	
**open**	0.071*** [0.008]		0.036*** [0.079]		0.021*** [0.011]	
**govrise**	0.004 [0.003]		0.004 [0.003]		0.008 [0.004]	
**Control variables**	yes		yes		yes	
**Constant terms**	yes		yes		yes	
**Observed values**	672		672		672	
**Robust(R²)**	0.6637		0.9674		0.8564	

Note: *, ** and *** are significant at 10%, 5% and 1%, respectively, with data as standard error in parentheses.

To ensure the consistency and unbiased nature of our estimation results, we conducted a series of validity tests for the instrumental variables, including the LM test, the test for over-identification, and the test for weak instruments. As shown in Table 5, all tests yielded p-values below 0.001, confirming the high correlation between the instrumental variables and the endogenous explanatory variables, with no correlation with the error term, thus satisfying the relevance assumption. Additionally, the absence of weak instruments ensures the consistency of our estimates. These findings validate our choice of the IV-2SLS method and suggest that the endogeneity issue has been effectively addressed.

**Table 5:** Validity test results of tool variables.

	LM	Over identification	Weak recognition
**L.Lnndp**	1406.22 [0.000]	1586.29 [0.000]	1562.68
**L.VFI**	555.01 [0.000]	734.26 [0.000]	723.33
**L.open**	2085.31 [0.000]	1657.35 [0.000]	1632.68
**L.govrise**	735.30 [0.000]	2081.59 [0.000]	2050.62

#### 5.1.2 Dynamic panel model—GMM regression

Given the notable carryover effect in real-world income inequality among residents, this study incorporates the lagged term of the Gini coefficient into the equation, as suggested by scholars [62]. A dynamic panel model, presented in Eq (4), is constructed to conduct an empirical examination of how vertical fiscal imbalance affects income inequality among residents.


$$Gini_{it}=\lambda_{j}\sum_{j=1}^{p}Gini_{it-j}+\partial_{1}VFI_{it}+\emptyset X_{it}+\varepsilon_{it}$$ (4)

This study uses the differential GMM and system GMM approaches to estimate Eq (4). There might be a two-way causal relationship between the explanatory variables, such as vertical fiscal imbalance, economic development level, and the Gini coefficient. The lagged values of the dependent variable and other explanatory variables could lead to endogeneity issues. These empirical methods are employed to handle these problems effectively. Moreover, to prevent over-identification of instrumental variables, the lag orders of the dependent variable and endogenous explanatory variables are restricted during the selection of instrumental variables.

In column (7), the differential GMM estimation uses lags 2 to 4 of the Gini coefficient and includes vertical fiscal imbalance, financial development level, and population density as exogenous control variables. To ensure the robustness of the empirical results, stricter exogenous variables are chosen as instrumental variables for the model; therefore, column (8) uses the system GMM estimation with the population natural growth rate as the exogenous instrumental variable.

Considering that government competition is influenced by factors such as resident income and economic development level, column (9) uses the differential GMM estimation with lags 2 to 4 of the Gini coefficient, vertical fiscal imbalance, financial development level, human capital, and government competition as exogenous control variables. Considering the structural bias of fixed capital investment under the background of local government fiscal imbalance, column (10) incorporates physical capital as an instrumental variable in the system GMM estimation. Table 6 reports the GMM regression results.

**Table 6:** Results of the GMM regression.

Variable	(7) Fd-gmm	(8) Sys-gmm	(9) Fd-gmm2	(10) Sys- gmm2
**L.Gini**	0.212** (-0.099)	0.392** (-0.196)	0.205** (-0.09)	0.519*** (-0.053)
**VIF**	0.027*** (-0.009)	0.171* (-0.094)	0.013* (-0.007)	0.108** (-0.054)
**lngdp**	-0.087*** (-0.031)	-0.057* (-0.031)	-0.013** (-0.006)	-0.014*** (-0.003)
**open**	0.089*** (-0.033)	0.137 (-0.086)	-0.002 (-0.025)	0.039* (-0.022)
**govrise**	0.821** (-0.408)	-0.322 (-0.443)	0.074 (-0.055)	0.26 (-0.176)
**financial**	-0.207* (-0.119)	1.576 (-1.198)	-0.228** (-0.104)	-0.118 (-0.095)
**phycap**	-0.023** (-0.01)	-0.035** (-0.017)	0.011 (-0.009)	-0.009 (-0.009)
**hucap**	0.016*** (-0.006)	0.030 (-0.02)	0.028*** (-0.004)	0.016** (-0.007)
**Urban**	0.189** (-0.074)	-0.350* (-0.208)	0.074** (-0.029)	-0.066** (-0.03)
**density**	-0.001 (-0.001)	0.034*** (-0.011)	-0.012*** (-0.003)	0.006 (-0.005)
**N**	644	672	644	672
**AR (2)**	0.906	0.875	0.621	0.833
**Sargan-Test**	0.480	0.646	0.451	0.328
**Hansen-Test**	0.719	0.844	0.327	0.680

Note: *, * * and * * * are significant at 10%, 5% and 1%, respectively, with data as standard error in parentheses.

The basic regression outcomes indicate that an escalation in vertical fiscal imbalance is conducive to an augmentation in the disparity of income distribution among urban and rural residents. A regression analysis conducted after the decomposition of the Gini coefficient reveals that an elevation in vertical fiscal imbalance exerts a notable positive influence on income inequality within both urban and rural sectors. The monotonous correlation between vertical fiscal imbalance and income inequality exhibits homogeneity across urban and rural demographics. The GMM regression results presented in Table 6 reinforce the hypothesis 1. Moreover, the lagged Gini coefficient results confirm the presence of significant carryover effects in income inequality between these two groups.

### 5.2. The threshold effect of public expenditure structure

#### 5.2.1 Setting of the threshold model

This study develops a threshold model for various public expenditure items to explore the threshold effects of public spending, thereby providing empirical support for Hypotheses 2 and 3. Fiscal instruments, particularly the structure of public expenditures, play a crucial role in influencing the income disparity between urban and rural populations. A rigid public expenditure structure can limit the impact of vertical fiscal imbalance, thereby alleviating its negative effects on income distribution. To fully understand the role of vertical fiscal imbalance in income distribution, it is essential to examine the structure of public expenditures and the urban-rural divide.

The research employs the threshold model [63], treating the threshold value as an unknown variable and constructing piecewise functions for different public expenditure items to analyze the threshold value and its effects. Additionally, the study estimates threshold models for the income Gini coefficients of the general population, urban residents, and rural residents, each in relation to five types of public expenditure structures.

This approach is adopted for two primary reasons: Firstly, the components of public expenditure exhibit a structural interdependence characterized by mutual restriction, and focusing solely on the overall scale of public expenditure would disrupt the structural relationships between different expenditure components. Secondly, it is imperative to consider the functional attributes of various expenditures, as distinct expenditure structures can exert diverse impacts on income distribution. To facilitate clarity, multiple threshold models are consolidated and represented as Eq (5).


$$\begin{array}{c} Gini_{it}=\partial_{0}+\beta_{1}VFI_{it}\cdot I(q_{it}⩽\gamma )+\beta_{2}VFI_{it}\cdot I(\gamma <q_{it})+\partial_{1}lngdp_{it}\\ +\partial_{2}open_{it}+\partial_{3}govrise_{it}+\partial_{4}finance_{it}+\partial_{5}phycap_{it}\\ +\partial_{6}hucap_{it}+\partial_{7}urban_{it}+\partial_{8}density_{it}+u_{i}+\varepsilon_{it} \end{array}$$ (5)

In the context of this model, *n* denotes provinces and *i* denotes years. The dependent variable (*Gini*_*it*_) encompasses three categories of Gini coefficients: the Gini coefficient for urban resident income ($Gini_{it}^{u}$), the Gini coefficient for rural resident income ($Gini_{it}^{r}$), and the Gini coefficient for national resident income ($Gini_{it}^{g}$). The variable *q*_*it*_ signifies the threshold variable for public expenditure structures, encompassing the proportions of expenditures on SECHS (*q*_1_), AFW (*q*_2_), Public Safety (*q*_3_), Social Security (*q*_4_), Social Security and Employment (*q*_5_).*VFI*_*it*_ Represents the core explanatory variable influenced by the threshold variable, γ denotes the unknown threshold value of public expenditure, and *β*_2_ correspond to the coefficients of variable v when the threshold variable *q*_*it*_ is less than γ and greater than γ, respectively. *I*(⋅) denotes the indicator function, *u*_*it*_ represents the regional non-observed effect, and ε_*it*_ indicates the rand *β*_1_ om error.

#### 5.2.2 Test of the threshold effect

This study investigates the threshold effect in public expenditure configurations amid escalating vertical fiscal imbalances and their impact on income inequality. Analyses were conducted across spending categories including SECHS (science, education, culture, health, and sports), AFW (agriculture, forestry, water, and hydropower), public safety, social security, employment, and public administration.

Significant findings are presented in Table 7, which includes the F-statistic, probability P-values, and confidence intervals at the 10%, 5%, and 1% levels, along with the threshold values. These values were calculated using a grid search method with 400 grid points, excluding 1% of outliers, and the bootstrap method was employed for 300 iterations to test the significance of the threshold effects.

**Table 7:** Test of the threshold effect of public expenditure.

Threshold variable	Dependent variable	Threshold number	F-value	P -value	10%	5%	1%	Threshold value
**SECHS**	Urban	Single	36.07**	0.0467	30.16	33.43	42.59	0.0800
		Double	36.86***	0.0100	24.55	29.64	36.54	0.0248
**AFW**	Urban	Single	31.27**	0.0367	35.21	39.14	55.32	0.0167
	Rural area	Single	39.31*	0.0733	26.68	32.57	40.78	0.0080
**Public Safety**	Urban	Single	36.51*	0.0800	27.19	29.73	37.13	0.0580
		Double	46.99***	0.0033	36.00	42.88	78.54	0.0068
**Social Security**	Nationwide	Single	61.50***	0.0067	33.41	42.11	59.65	0.0166
	Urban	Single	49.36***	0.0100	32.60	40.13	49.00	0.0071
**Administration**	Nationwide	Single	49.36**	0.0233	36.95	45.26	52.83	0.0072
	Urban	Single	43.93***	0.0067	27.13	33.36	41.74	0.0061
	Rural area	Single	31.79*	0.0900	31.12	35.34	51.82	0.0070

Note: *, * * and * * * are significant at 10%, 5% and 1%.

The results reveal a pronounced threshold effect of the public expenditure structure on the relationship between vertical fiscal imbalance and income inequality among Chinese residents. For urban income inequality, SECHS expenditures validated the single threshold model at the 5% and 10% significance levels, and the dual threshold model at the 1% level. AFW spending, below 0.0580% of the budget, has a reduced positive impact on income inequality, which increases stepwise with rising expenditures. In the sectors of public safety, social security, and administrative management, the single threshold model was validated at varying significance levels. Rural income inequality showed validation of the single threshold model at the 10% significance level for corresponding expenditures. National income inequality analyses revealed that social security and administrative management expenditures validated the single threshold model at the 1% and 5% significance levels, respectively.

### 5.3. Analysis of the threshold test results

#### 5.3.1 Inequality of urban residents

The threshold estimates presented in Table 8 reveal a complex, non-linear relationship between vertical fiscal imbalances and urban income inequality. Specifically, SECHS (Science, Education, Culture, Health, and Sports) expenditures below 0.0248% of the total budget have no discernible effect on income inequality. However, beyond this threshold, each 1% increase in fiscal imbalance corresponds to a 9.8% rise in income inequality, a finding supported by recent research [64], which identified a similar threshold effect on the urban-rural income gap.

**Table 8:** Estimating the threshold of public expenditure structure for urban residents.

The threshold variable		SECHS	AFW	Public safety	Social security	Administration
Threshold value	γ1	0.0248	0.0580	0.0080	0.0071	0.0061
	γ2	0.0800	——	0.0167	——	——
VIF	Zone system1	0.010	0.061***	0.010	-0.024*	-0.074***
	(*q*_*i*_ ≤ γ1)	-0.015	-0.013	-0.014	-0.014	-0.023
	Zone system2	0.055***	0.104***	0.049***	0.039***	0.059***
	(γ1 < *q*_*i*_ ≤ γ2)	-0.012	-0.015	-0.012	-0.012	-0.013
	Zone system3	0.098***	——	0.089***	——	——
	(γ2 < *q*_*i*_)	-0.014		-0.014		
Economic development level		-0.002	0.001	-0.004	0.004	0.005
		(-0.006)	(-0.006)	(-0.006)	(-0.006)	(-0.006)
Open to the outside world		0.032***	0.034***	0.034***	0.037***	0.026***
		(-0.009)	(-0.009)	(-0.009)	(-0.009)	(-0.009)
The government competition		0.057	0.087	0.048	-0.01	-0.086
		(-0.074)	(-0.076)	(-0.074)	(-0.073)	(-0.08)
Financial development level		-0.228***	-0.130*	-0.161**	-0.068	-0.171**
		(-0.077)	(-0.077)	(-0.075)	(-0.073)	(-0.077)
Material capital		-0.016**	-0.017**	-0.019**	-0.006	0.000
		(-0.007)	(-0.008)	(-0.007)	(-0.007)	(-0.008)
Human capital		0.021***	0.031***	0.022***	0.020***	0.027***
		(-0.004)	(-0.003)	(-0.003)	(-0.003)	(-0.003)
Urbanization rate		0.054***	0.058***	0.055***	0.042***	0.035***
		(-0.012)	(-0.012)	(-0.012)	(-0.012)	(-0.012)
Density of population		0.001	0.002	0.000	0.000	0.003*
		(-0.002)	(-0.002)	(-0.002)	(-0.002)	(-0.002)
N		700	700	700	700	700
R^2^		0.45	0.415	0.46	0.47	0.43

Note: *, * * and * * * are significant at 10%, 5% and 1%, respectively, with data as standard error in parentheses.

Public safety expenditures exhibit a similar pattern, with no significant impact at lower levels but a nearly doubled positive impact at higher levels, aligning with the findings of the study [65]. AFW (Agriculture, Forestry, Water, and Hydropower) spending, below 0.0580% of the budget, has a reduced positive impact on income inequality, which increases stepwise with rising expenditures, consistent with the research [66] on the differential impacts of fiscal expenditure structure. Social security expenditures alleviate fiscal imbalance’s negative impact on urban income distribution at lower levels but exacerbate inequality at higher levels. Similarly, administrative expenditures counteract fiscal imbalance’s effects initially but become counterproductive beyond a threshold. Both types of expenditures exhibit a “U”-shaped influence on urban income distribution.

Therefore, it is recommended to refine the fiscal expenditure structure by establishing budget thresholds for various categories of spending, progressively enhancing public security allocations, optimizing expenditures in agriculture, forestry, and water conservancy, prioritizing social security outlays, prudently managing administrative expenses, and fostering an inter-regional coordination mechanism. These initiatives are poised to foster equitable income distribution and mitigate the adverse effects of fiscal imbalances on urban income disparities.

#### 5.3.2 Inequality in income among rural residents

The threshold estimation results presented in Table 9 (Columns 2–3) reveal a significant asymmetry in the impact of vertical fiscal imbala reveal a significant asymmetry in the impact of vertical fiscal imbalance on rural income inequality. For AFW expenditures, in the low-expenditure zone (system 1), a 0.054% rise in rural income inequality is associated with each 1% increase in fiscal imbalance, consistent with the research [67] demonstrating how insufficient spending in sectors like science education and agriculture can exacerbate the negative effects of fiscal imbalance. This remains the case despite overall increases in local government expenditures due to fiscal decentralization. Moreover, upon exceeding the 0.0068 threshold and entering high-expenditure zone (system 2), the negative impact is notably diminished, aligning with the research [64] that identifies an inverted U-shaped relationship between fiscal bias and the urban-rural income gap, indicating a potential reduction in the income gap beyond a certain threshold of fiscal bias.

**Table 9:** Results of threshold estimates for public expenditure for rural and national residents.

		Rural area		Nationwide	
The threshold variable		AFW	Administration	Social security	Administration
threshold value		0.0068	0.0070	0.0166	0.0072
VIF	Zone system1 ((*q*_*i*_ < γ1))	0.054*** (-0.019)	0.086*** (-0.023)	0.022* (-0.013)	0.006 (-0.015)
	Zone system2 ((γ < *q*_*i*_))	-0.004 (-0.017)	-0.010 (-0.017)	0.058*** (-0.011)	0.071*** (-0.011)
Economic development level		-0.017* (-0.009)	-0.021** (-0.009)	-0.023*** (-0.006)	-0.018*** (-0.006)
Open to the outside world		0.002 (-0.012)	0.010 (-0.012)	0.026*** (-0.008)	0.021*** (-0.008)
The government competition		0.021 (-0.101)	0.144 (-0.106)	0.191*** (-0.067)	0.153** (-0.069)
Financial development level		-0.119 (-0.103)	-0.116 (-0.103)	-0.160** (-0.067)	-0.222*** (-0.068)
Material capital		0.026** (-0.01)	0.015 (-0.01)	-0.027*** (-0.007)	-0.015** (-0.007)
Human capital		0.016*** (-0.005)	0.018*** (-0.005)	0.036*** (-0.003)	0.039*** (-0.003)
Urbanization rate		0.023 (-0.017)	0.028 (-0.017)	-0.022** (-0.01)	-0.050*** (-0.011)
Density of population		-0.002 (-0.002)	-0.002 (-0.002)	-0.001 (-0.001)	0.001 (-0.001)

Note: *, * * and * * * are significant at 10%, 5% and 1%, respectively, with data as standard error in parentheses.

Furthermore, the findings suggest that fiscal vertical imbalances might exacerbate the rural income disparity when the ratio of administrative expenditure to total expenditure drops below 0.007. This phenomenon could be attributed to factors such as a lagging rural economy, low educational levels, complex management structures, and limited efficiency in the implementation of artificial intelligence. In contrast to the perspective posited by other scholars. For instance, Fuyun Jie et al. [66] argued that escalating administrative spending could exacerbate this disparity, while scholars [58, 68] specifically underscored a particular focus on rural regions.

Therefore, increasing spending on agriculture, forestry, water resources and hydropower and reasonably increasing administrative expenditure can help prevent further widening of the income gap in rural areas.

#### 5.3.3 Income inequality across the country

Table 9 (Columns 4–5) reveals a significant association between vertical fiscal imbalance and overall income inequality, with a pronounced differential effect based on varying levels of social security and administrative spending. Below a threshold of 0.0166 in social security expenditure relative to the total budget, a 1% increase in fiscal imbalance is associated with a 0.022% increase in income inequality. However, exceeding this threshold amplifies the impact, resulting in a 0.058% rise in income inequality for each 1% increase in fiscal imbalance. This non-linear, stepwise growth pattern is in alignment with the research [69, 70] documenting progressive effects of social spending. Furthermore, when administrative expenses exceed 0.0072 of the total budgets, the income gap widens by 0.071% for every 1% increase in fiscal imbalance.

This section emphasizes the threshold effects of social security and administrative expenses on national income inequality, representing a broader measure of income disparity. In comparison, the previous two sections focused on the income gaps specific to urban and rural residents, allowing for more targeted policy interventions for different groups. The insights provided in this section are particularly valuable for addressing overall income inequality. By integrating the findings from all three sections, we gain a comprehensive understanding of the components of the overall effect, which is especially valuable for structural adjustment and policy formulation.

## 6. Conclusion

### 6.1. Main findings

It is concluded in this research that fiscal vertical imbalance significantly exacerbates income inequality, a finding supported by the studies [26, 33, 71].

Additionally, a threshold effect in public spending is identified, signifying non-linear impacts on income disparity. This conclusion is consistent with the advocacy for balanced urban-rural development and rural infrastructure investment by scholars [32, 65, 72], who highlight the adverse effects of insufficient spending in vital sectors such as agriculture and education on income inequality.

### 6.2. Unique contributions and geographic specificity

Our study uniquely identifies the varying threshold effects of urban public expenditure on the income disparities between rural areas, urban areas, and the gap between them. This geographic specificity is also emphasized by scholars [65, 73], who discuss the regional dimensions of fiscal policy effectiveness in their respective studies. Scholars [64, 74, 75] highlight an inverted U-shaped relationship between fiscal imbalance and the urban-rural income gap, underscoring the importance of fiscal thresholds. Our focus on the threshold effect’s dependence on public expenditure structure and its variation between regions adds geographic specificity to understanding fiscal impacts on income inequality.

### 6.3. Policy recommendations

The findings suggest important policy recommendations: Firstly, it is necessary to increase investment in rural contemporary infrastructure, including agriculture, forestry, water conservancy, and transportation [35]. Secondly, this paper agrees with the perspective [32] of increasing fiscal expenditure in rural areas, science, education, culture, and health can effectively reduce the urban-rural income gap. However, based on the empirical results of this paper, spending on science, education, culture, and health exacerbates the income gap among urban residents, while its effect on the rural income gap and the urban-rural income gap is not significant. In addition, this paper suggests fully considering the fiscal deficit situation and reasonably controlling social security and employment expenditures, especially urban social security expenditures. Lastly, reducing management expenditures in well-served city centers and reallocating funds to rural areas in urgent need is consistent.

## 7. Limitations and future research

### 7.1. Study limitations

Our study is subject to several inherent limitations due to the scope of the data and the unique context of our analysis. Firstly, the sample size is limited to the provincial level, which may constrain the generalizability of our findings. A more extensive sample, incorporating municipal or county-level data, could provide a richer understanding of the relationship between public expenditure, the threshold effect, and income inequality. However, given the scope and resources of our current study, we acknowledge that such an expansion was beyond our immediate capabilities. Secondly, language expression poses a limitation, as it may affect the rigor of our discourse. We have strived for clarity and precision in our articulation, but the subtleties of language could potentially impact the exactness of our explanations and conclusions, leading to potential uncertainties in interpretation. Thirdly, our literature review and comparative analysis are predominantly focused on studies within the context of China’s unique national conditions. This focus, while relevant for understanding the domestic context, may limit the breadth of our comparative analysis and the generalizability of our findings to other countries with different socio-economic and political landscapes.

### 7.2. Future work plans

Acknowledging the limitations of our study, we suggest that future research should particularly consider the potential urban-rural differences in the threshold effects of various expenditure items. While our current analysis provides a general understanding of the relationship between public expenditure and income inequality at the provincial level, it does not fully account for the distinct economic and social contexts of urban and rural areas. Exploring these differences could reveal important nuances in how different types of public spending affect income distribution across diverse geographic and demographic settings. Such an exploration is crucial for developing more targeted and effective fiscal policies that address the unique challenges of both urban and rural populations.
